# Global trends and hotspots in the digital therapeutics of autism spectrum disorders: a bibliometric analysis from 2002 to 2022

**DOI:** 10.3389/fpsyt.2023.1126404

**Published:** 2023-05-15

**Authors:** Xuesen Wu, Haiyin Deng, Shiyun Jian, Huian Chen, Qing Li, Ruiyu Gong, Jingsong Wu

**Affiliations:** ^1^College of Rehabilitation Medicine, Fujian University of Traditional Chinese Medicine, Fuzhou, Fujian, China; ^2^Innovation and Transformation Center, Fujian University of Traditional Chinese Medicine, Fuzhou, Fujian, China

**Keywords:** autism, digital therapeutics, CiteSpace, bibliometrics, knowledge mapping

## Abstract

**Introduction:**

Autism spectrum disorder (ASD) is a severe neurodevelopmental disorder that has become a major cause of disability in children. Digital therapeutics (DTx) delivers evidence-based therapeutic interventions to patients that are driven by software to prevent, manage, or treat a medical disorder or disease. This study objectively analyzed the current research status of global DTx in ASD from 2002 to 2022, aiming to explore the current global research status and trends in the field.

**Methods:**

The Web of Science database was searched for articles about DTx in ASD from January 2002 to October 2022. CiteSpace was used to analyze the co-occurrence of keywords in literature, partnerships between authors, institutions, and countries, the sudden occurrence of keywords, clustering of keywords over time, and analysis of references, cited authors, and cited journals.

**Results:**

A total of 509 articles were included. The most productive country and institution were the United States and Vanderbilt University. The largest contributing authors were Warren, Zachary, and Sarkar, Nilanjan. The most-cited journal was the *Journal of Autism and Developmental Disorders*. The most-cited and co-cited articles were Brian Scarselati (Robots for Use in Autism Research, 2012) and Ralph Adolphs (Abnormal processing of social information from faces in autism, 2001). “Artificial Intelligence,” “machine learning,” “Virtual Reality,” and “eye tracking” were common new and cutting-edge trends in research on DTx in ASD.

**Discussion:**

The use of DTx in ASD is developing rapidly and gaining the attention of researchers worldwide. The publications in this field have increased year by year, mainly concentrated in the developed countries, especially in the United States. Both Vanderbilt University and Yale University are very important institutions in the field. The researcher from Vanderbilt University, Warren and Zachary, his dynamics or achievements in the field is also more worth our attention. The application of new technologies such as virtual reality, machine learning, and eye-tracking in this field has driven the development of DTx on ASD and is currently a popular research topic. More cross-regional and cross-disciplinary collaborations are recommended to advance the development and availability of DTx.

## 1. Introduction

Autism spectrum disorder (ASD) is a childhood-onset neurodevelopmental disorder characterized by impaired social communication and repetitive behaviors (1–3). According to survey data, the global prevalence of ASD was 1% in 2021 (4) and continues to rise (5). Compared with neurotypical children, children with ASD require more care from their caregivers (6, 7). Due to atypical social interaction and communication, children with ASD bullied by peers often refuse to school (8). All of these place a heavy burden on the children and their families. The assessment and treatment of ASD is currently a medical challenge, and its treatment remains costly (9). New methods to diagnose and treat people with ASD more effectively are currently being explored.

With the advent of technology, digital therapeutics (DTx) have emerged to provide novel avenues to diagnose and treat ASD. In 2017, DTx were defined by the Digital Therapeutics Alliance as “evidence-based therapeutic interventions driven by high-quality software programs to prevent, manage, or treat a medical disorder or disease” (10). Digital Therapeutics Alliance also developed core principles and best practice guidelines related to design, manufacturing, clinical validation, and regulatory oversight (10), driving the development of DTx.

In 2021, the Canvas Dx developed by Cognoa became the first ASD diagnostic device to be authorized by the United States Food and Drug Administration (FDA) (11). In recent years, emerging technologies such as machine learning (ML) (12), virtual reality (VR) (13), face recognition (14), telemedicine (15), and wearable devices (16) have also been used in DTx for ASD. These technologies enhance the identification and measurement of behavioral, symptomatic, or biological factors associated with ASD (17), strengthen the therapeutic effect of DTx (18, 19), reduce treatment costs (20), and provide more flexibility to meet individual patient needs (21).

Digital therapeutics has been used in the screening, diagnosis and treatment of ASD. In the area of ASD screening and diagnosis, DTx can help analyze the characteristics and behaviors of children with ASD, provide ASD risk assessment for children, and assist clinicians in improving diagnostic efficiency and accuracy. A systematic review showed that artificial intelligence (AI)-assisted DTx diagnostic tools can be used for early screening and diagnosis of ASD (22). For example, questionnaires and home videos combined with ML algorithms to screen children with ASD were superior to common screening tools in terms of sensitivity and specificity (23). Eye-tracking technology combined with ML, which collects eye movement data by watching videos and analyzes visual attention models to aid in the diagnosis of ASD, showed an average accuracy and specificity of 90 and 93%, respectively (24). In terms of treatment, AI-based robots can provide adjunctive psychological interventions for social problems in children with ASD (25). Smartphones (26) and iPads (27) based on games or stories for training children with ASD are also effective and can reach a larger number of children. VR-based and computerized training programs that help create different life scenarios can also have a positive effect on improving emotional recognition in ASD (28). As DTx have been widely used in the field of ASD in recent years, a variety of new techniques, methods, and concepts have emerged. In order to help researchers to conduct deeper research or explore frontier research directions in DTx on ASD, it is necessary to sort them out more systematically to identify current research hotspots and future development trends. Bibliometrics is widely used to analyze published scientific literature. In recent years, bibliometric analyses have been conducted on the overall application status and research trends of DTx (29, 30). However, to the best of our knowledge, there is no literature analysis on the broad topic of DTx applications for ASD. Therefore, this study aimed to analyze research on DTx for ASD from 2002 to 2022 with the help of a literature visualization tool to provide a comprehensive analysis of research hotspots, future trends, challenges, and prospects.

## 2. Materials and methods

All data were obtained from the Web of Science (WoS) Core Collection on October 20, 2022. The relevant literature was published between January 1, 2002, and October 20, 2022, and the themes of the data search were “Digital Therapy” and “Autism Spectrum Disorder” (Supplementary Table 1). Literature search was constrained to articles and review articles, eliminating other document types. Search results were analyzed using CiteSpace (31).

Data collection and analysis were rescreened by Xuesen Wu, Shiyun Jian, Huian Chen, Qing Li, and Ruiyu Gong. Only literature related to DTx for ASD were included, and duplicates were removed. Any disagreements were settled through discussion or by asking for assistance from other authors. A total of 509 publications were included (Supplementary Figure 1), including 451 regular articles and 58 review articles (Supplementary Figure 2).

CiteSpace (6.1.R3) is a scientific literature visual analysis software developed in the context of scientometrics and data visualization. It presents the structure, laws, and distribution of subject knowledge in the form of scientific knowledge graphs, and can determine progress of a certain field and the current research frontiers (31). The current study performed co-occurrence network analysis, clustering, centrality analysis, and timeline analysis using CiteSpace. For this, time-slicing was performed from January 2002 to October 2022 (1 year per slice), and we set node type (1 per time), selection criteria (*g*-index: *k* = 25), and pruning (pathfinder, pruning sliced network). For the parameter settings of CiteSpace, we set the *K* value to 25, which affects the number of nodes in the visualization graph, and set *N*% to 10, which represents the top 10% of the most referenced or occurring items from each slice; default settings were used for other software values.

Each node is represented by a circle in the co-occurrence network graph formed by CiteSpace. The circle’s size shows the node’s frequency, and the greater the frequency, the larger the node. The circle’s color indicates the time when the node appears, and the darker the color, the earlier the node appears. And the purple ring represents the centrality of the node, which is meaningful when the centricity is greater than 0.1. Centrality indicates the degree of concern about the study findings. In the cluster plot, CiteSpace provides two metrics, the modular value (*Q* value) and the average contour value (*S* value), which can be used to judge the mapping effect based on the network structure and clustering clarity. In general, *Q* values > 0.3 indicate significant community structure, *S* values > 0.5 indicate reasonable overall clustering, and *S* values > 0.7 indicate that the clustering is convincing (32). As for the order of the clusters, the smaller the number, the more keywords are included, and each cluster is composed of multiple closely related words.

Furthermore, we calculated authors’ H-indexes per 2-year time periods. The H-index means that a researcher has H papers cited at least H times, which can more accurately reflect a person’s academic achievement (33). The higher the H-index, the greater the reach and influence of their published work.

## 3. Results

### 3.1. The quantity, citations, and annual trends of published literature

**Figure 1:**
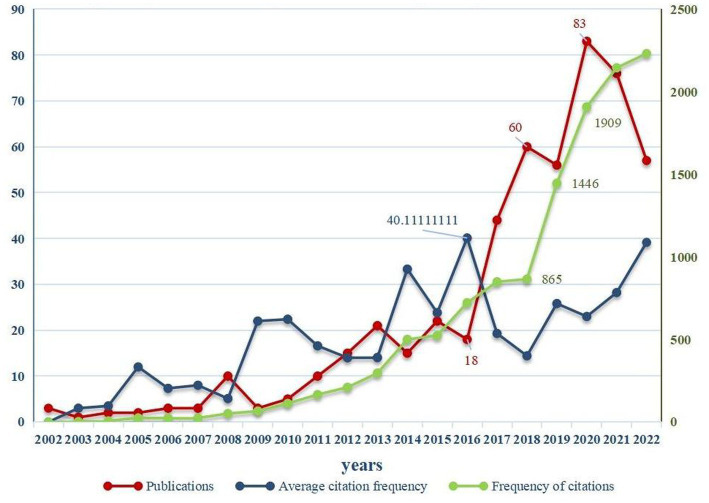
The annual publication, citation frequency and average citation frequency of bibliometrics related to digital therapeutics (DTx) on autism spectrum disorder (ASD) from 2002 to 2022.

A total of 509 records were included, with the number of publications broken down by year (Figure 1). The number of publications has increased over the past 20 years, with some fluctuations. Since 2002, the number of papers in this field has been growing, and the growth began to increase in 2010, with a surge in 2016 and a peak in 2020, which saw slightly lower annual growth until October 2022.

The annual citation frequency of articles has increased with the year (Figure 1), sharply in 2018–2020 and is growing steadily. Calculation showed that the average number of citations per paper was 23.916.

### 3.2. Analysis of cited journals

CiteSpace generated a map of cited journals (Supplementary Figure 3), generating 605 nodes and 2,728 links. The map’s nodes represent journals, and the linkages between them symbolize ties between co-citations. The top five periodicals with centrality were *Child Development* (0.39), *Archives of General Psychiatry* (0.22), *Acta psychiatrica Scandinavica* (0.22), *BRAIN* (0.2), and the *British Journal of Psychiatry* (0.15).

The top 10 journals ranked by citation frequency (Table 1) were selected, among which the *Journal of Autism and Developmental Disorders* was cited the most at 409, and 188 journals were cited more than 10 times.

**Table 1:** Top 10 frequency of cited journals related to digital therapeutics (DTx) on autism spectrum disorder (ASD).

Rank	Frequency	Cite journal	Rank	Frequency	Cite journal
1	409	Journal of Autism and Developmental Disorders	6	145	PLOS ONE
2	256	AUTISM	7	123	International Journal of Social Robotics
3	215	Research in Autism Spectrum Disorders	8	109	PEDIATRICS
4	204	Journal of Child Psychology and Psychiatry	9	102	Journal of the American Academy of Child and Adolescent Psychiatry
5	176	Autism Research	10	95	Research in Developmental Disabilities

Because the five selected bibliometrics described a non-normal distribution, we reported the median, quartile, and IQR (Supplementary Table 2). Most of the journals belonged to Q1, and the impact factor of more than half of the cited journals was above 4.489. The median Eigenfactor was 0.0143, the median CiteScore was 6.750, and the median source-normalized impact per paper and Scimago Journal Rank were 2.006 and 1.292, respectively. The range of influencing factors was 1–15. The Eigenfactor score of most journals was lower than 0.0221, and the CiteScore of most journals was between 5.850 and 9.825. Source-normalized impact per paper values were mainly between 1.560 and 2.944. The Scimago Journal Rank range was 0–4.

### 3.3. Analysis of source nations

**Figure 2:**
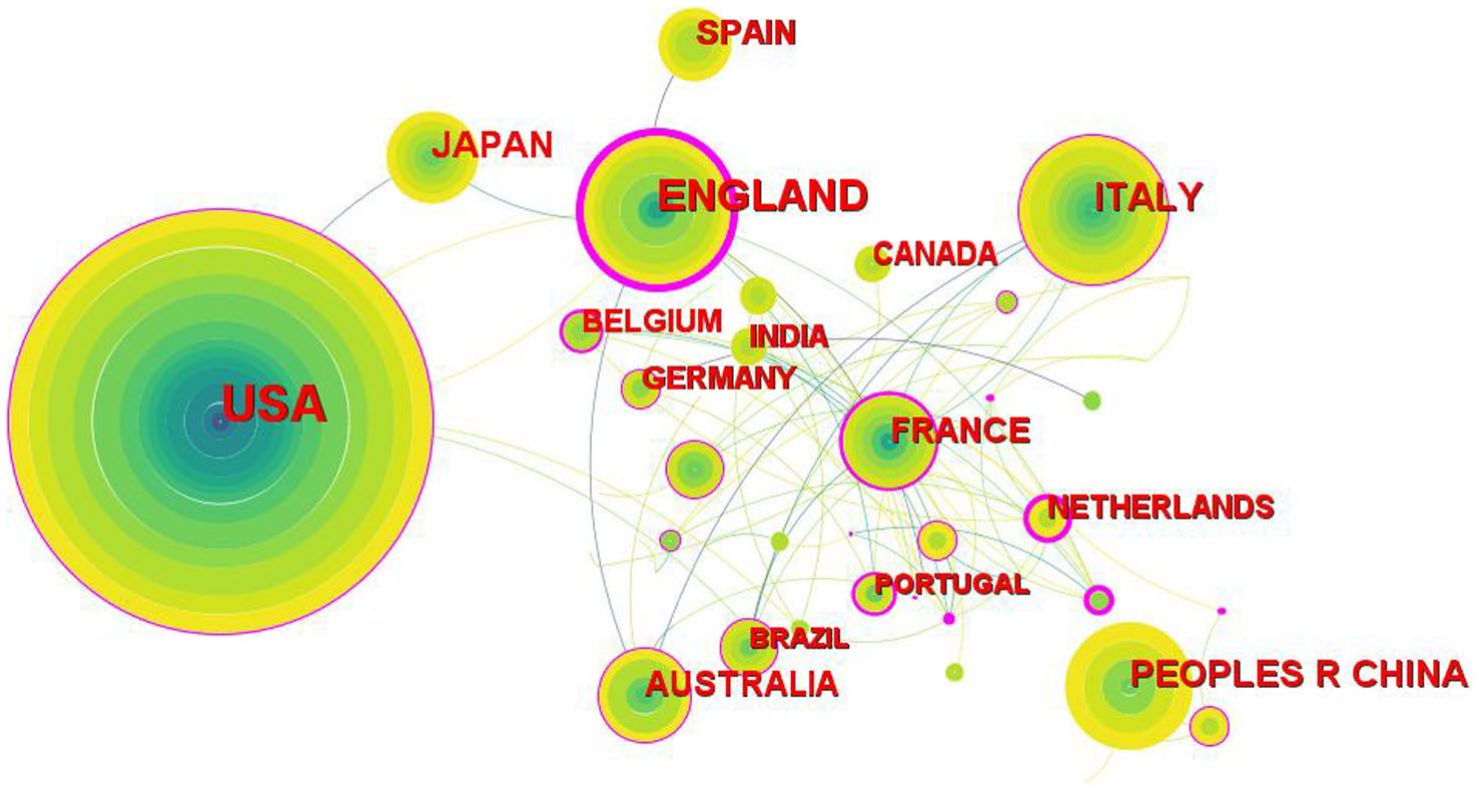
The collaboration network of countries related DTx on ASD.

Following the national visual analysis, 62 nodes and 82 linkages were generated on the maps (Figure 2), and the 509 publications that were included were written by academics from 61 different nations. In Table 2, which lists the top five countries for publishing, the United States of America (USA) was the major contributor, accounting for one-third of all articles (163) and being one of the first nations to publish these types of papers. The remaining top four countries were England (65), Italy (34), China (35), and Japan (32). In Figure 2, nodes represent countries. The top five countries in the center are England (0.76), Sweden (0.6), Singapore (0.58), Netherlands (0.54), and Belgium (0.34).

**Table 2:** Top 10 frequency and centrality of countries related to DTx on ASD.

Rank	Frequency	Country	Year	Centrality	Country	Year
1	163	United States	2002	0.76	England	2002
2	65	England	2002	0.6	Sweden	2013
3	44	Italy	2008	0.58	Singapore	2013
4	34	China	2011	0.54	Netherlands	2010
5	32	Japan	2005	0.34	Belgium	2013
6	26	France	2005	0.33	Turkey	2017
7	26	Australia	2008	0.29	France	2005
8	24	Spain	2004	0.27	Portugal	2013
9	21	Netherlands	2010	0.23	Egypt	2020
10	19	Belgium	2013	0.2	Brazil	2008

### 3.4. Analysis of authors

**Figure 3:**
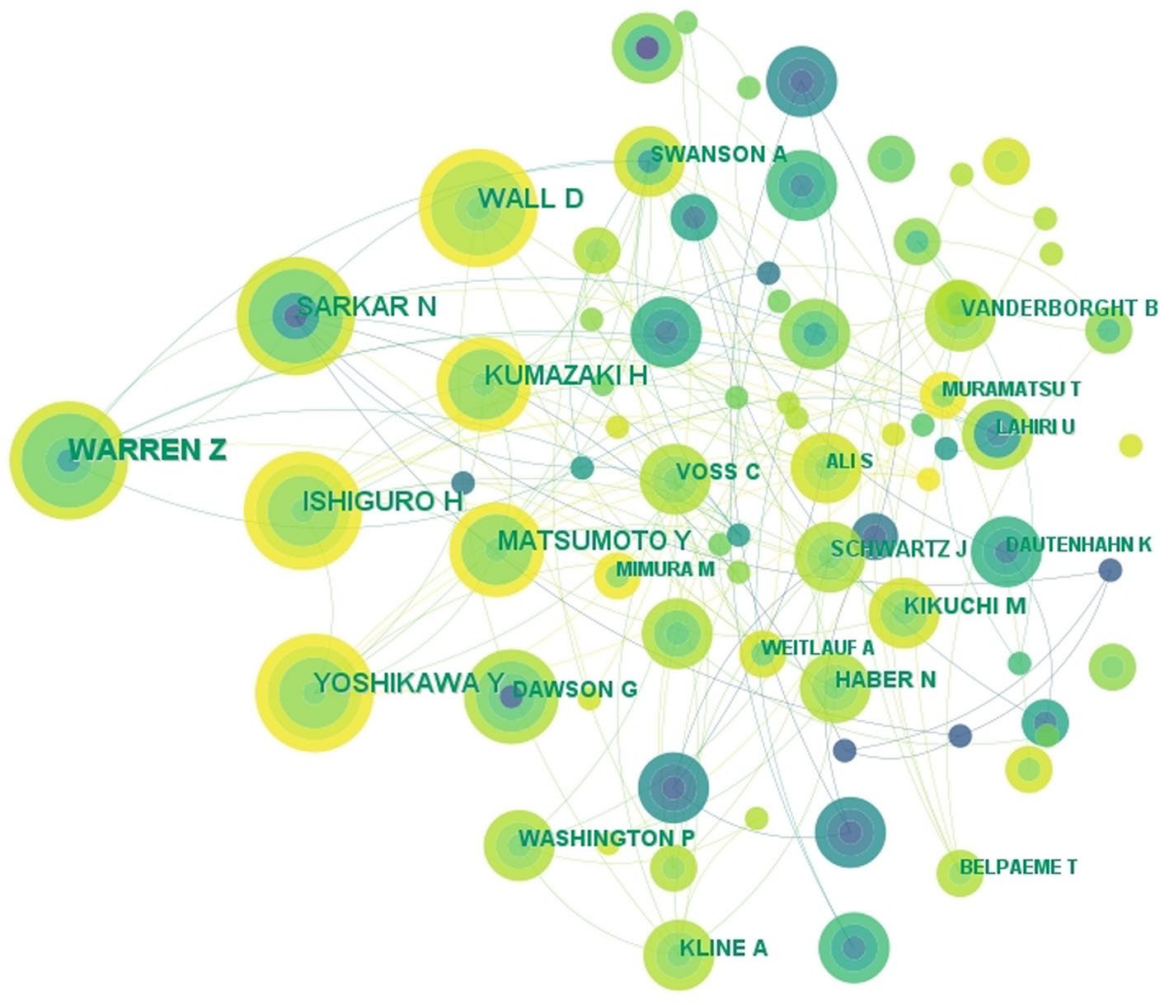
The collaboration network of authors related to DTx on ASD.

In the retrieved articles, the network graph formed with CiteSpace, consisting of 454 nodes and 743 links, indicated that these articles were published by 454 authors (Figure 3). The top five authors are Warren, Zachary (13), Sarkar, Nilanjan (11), Ishiguro, Hiroshi (11), Yoshikawa, Yuichiro (11) and Wall, Dennis P. (11). The total number of citations (Supplementary Figure 4) of the articles published by these authors was, respectively, 456, 511, 110, 110, and 249. The H-indexes per 2-year time period of papers published from these authors is found in Supplementary Figure 5.

### 3.5. Analysis of institutions

**Figure 4:**
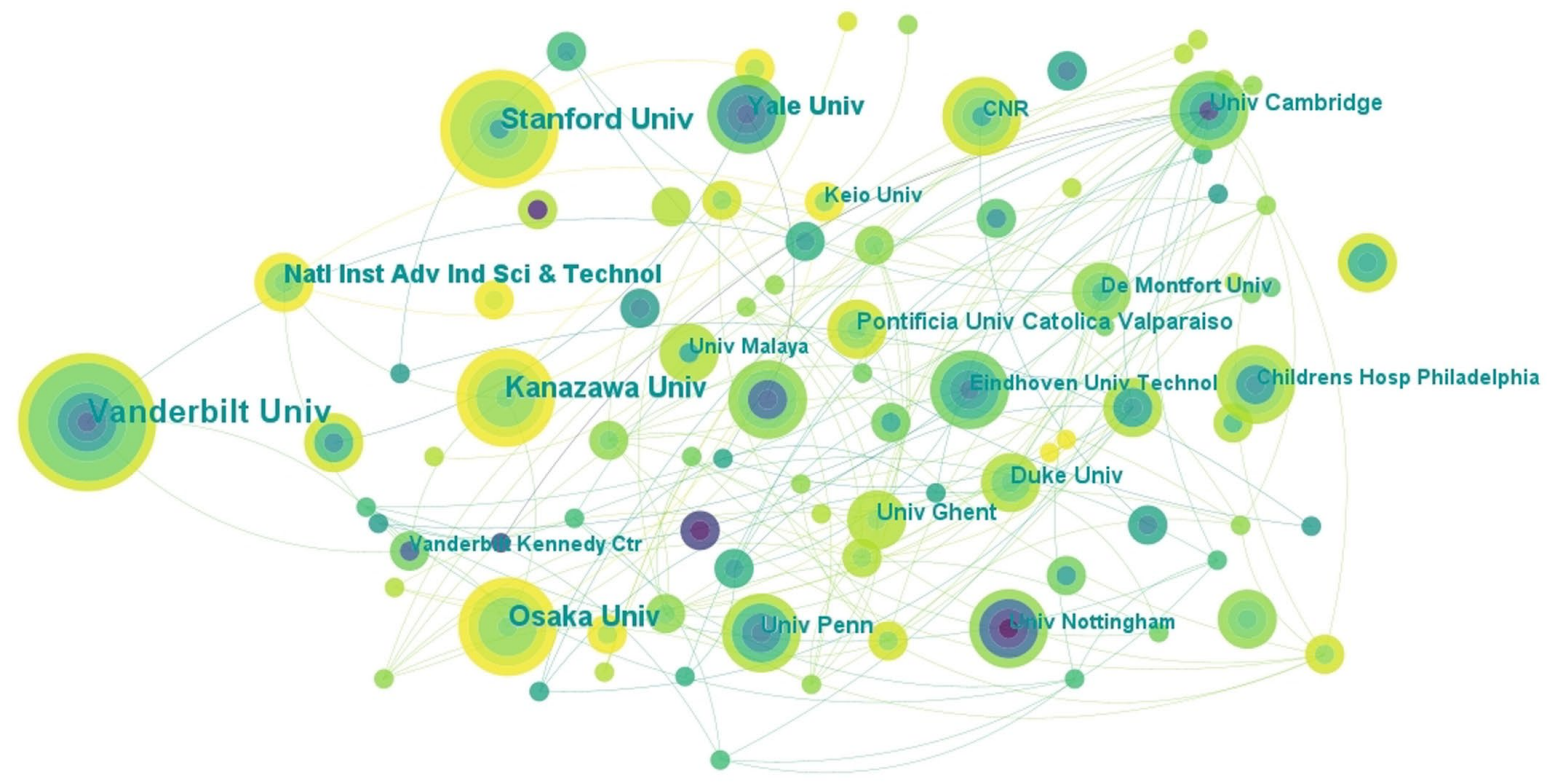
The collaboration network of institutions related to DTx on ASD.

A total of 360 institutions contributed to publications on DTx for ASD (Figure 4). The top five universities globally were Vanderbilt University (16), Stanford University (12), Kanazawa University (11), Osaka University (11), and Yale University (8).

### 3.6. Analysis of cited authors

**Figure 5:**
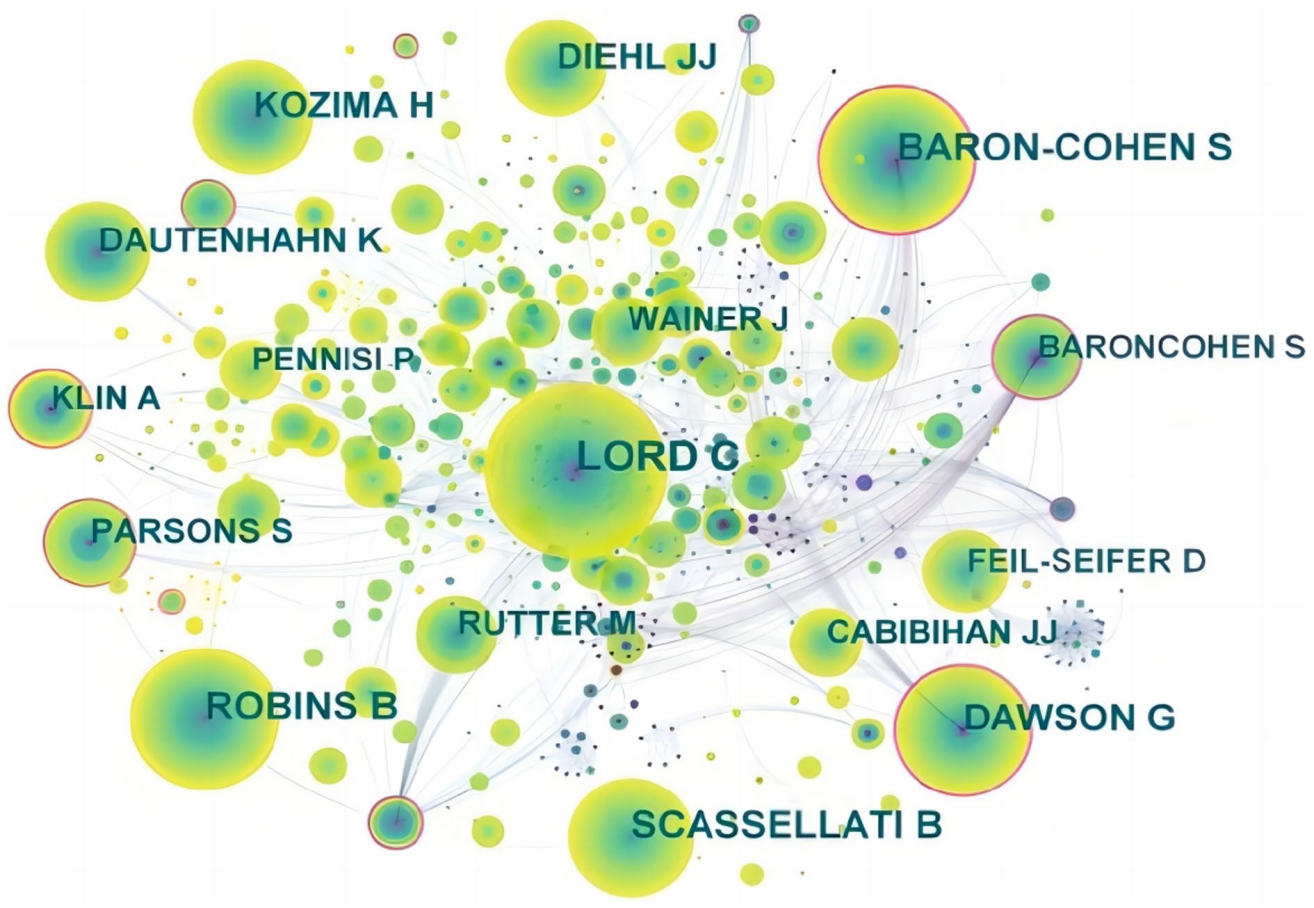
The citation network of authors related to DTx on ASD.

The map of the cited authors in Figure 5 shows that the top five most-cited authors were Lord Catherine, Robins Ben, Baron-Cohen Simon, Scassellati Brian, and Dawson Geraldine (Supplementary Table 3) from University of California, Los Angeles, Hertford University, Cambridge University, Yale University, and University of Washington, respectively.

Furthermore, as shown in Supplementary Table 3, the top five authors in terms of centrality were Billard Anais (0.35), Baron-cohen Simon (0.33), Baroncohen Simon (0.29), Dawson Geraldine (0.24), and Klin Ami (0.18). Because Baron-cohen Simon and Baroncohen Simon represent the same author, combining the centrality of them showed that this author had the highest centrality of the cited authors at 0.62. The author was later known as Baron-Cohen S.

### 3.7. Analysis of references

A co-citation map of the cited references (Supplementary Figure 6), and it contained 742 nodes and 2,296 linkages. The top 10 articles that cite sources are listed in Table 3. In terms of citation frequency, the top five articles were published by American Psychiatric Association in 2013 (86), Scassellati, Brian in 2012 (73), Diehl, Joshua J in 2012 (67), Catherine Lord in 2000 (36), and John-John Cabibihan in 2013 (36). Among the centrality of the cited references, the top five articles were published by Ralph Adolphs in 2001 (0.25), Feil-Seifer, David in 2009 (0.18), American Psychiatric Association in 2000 (0.18), Sarah Parsons in 2002 (0.16), and Giorgio Celani in 1999 (0.16).

**Table 3:** The top 10 cited articles related to the DTx on ASD.

Rank	The title of article	Year	Cited number	Journal	Impact factor
1	Diagnostic and statistical manual of mental disorders: DSM-5	2013	86	–	–
2	Robots for Use in Autism Research	2012	73	Annual Review of Biomedical Engineering	11.324
3	The Clinical Use of Robots for Individuals with Autism Spectrum Disorders: A Critical Review.	2012	67	Research in Autism Spectrum Disorders	3.293
4	The autism diagnostic observation schedule-generic: a standard measure of social and communication deficits associated with the spectrum of autism.	2000	42	Journal of Autism and Developmental Disorders	4.345
5	Why Robots? A Survey on the Roles and Benefits of Social Robots in the Therapy of Children with Autism	2013	42	International Journal of Social Robotics	3.802
6	Autism Diagnostic Interview-Revised: a revised version of a diagnostic interview for caregivers of individuals with possible pervasive developmental disorders.	1994	41	Journal of Autism and Developmental Disorders	4.345
7	Autism and social robotics: A systematic review	2016	39	Autism Research	4.633
8	Keepon: A Playful Robot for Research, Therapy, and Entertainment	2009	30	International Journal of Social Robotics	3.802
9	Prevalence of Autism Spectrum Disorder Among Children Aged 8 Years—Autism and Developmental Disabilities Monitoring Network, 11 Sites, United States, 2014	2018	29	MMWR Surveillance Summaries	29.095
10	Social robots as embedded reinforcers of social behavior in children with autism.	2013	26	Journal of Autism and Developmental Disorders	4.345

**Figure 6:**
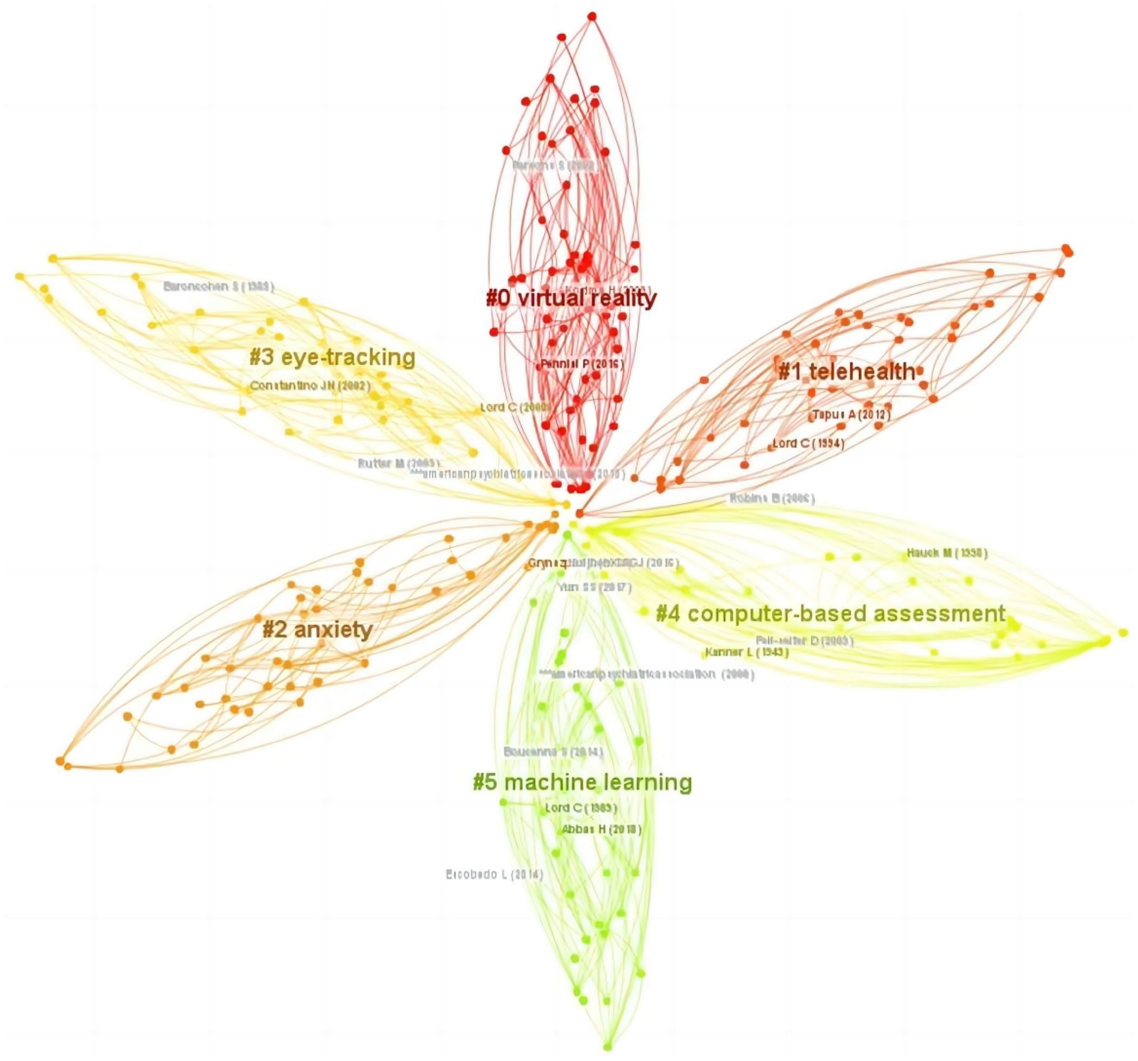
The cluster map of references related to DTx on ASD.

The cluster map of references is found in Figure 6. The *Q* value was 0.8975 and the *S* value was 0.9495, demonstrating a strong clustering effect. The top six clusters were “virtual reality,” “telehealth,” “anxiety,” “eye-tracking,” “computer-based assessment,” and “machine learning.”

### 3.8. Analysis of keywords

**Figure 7:**
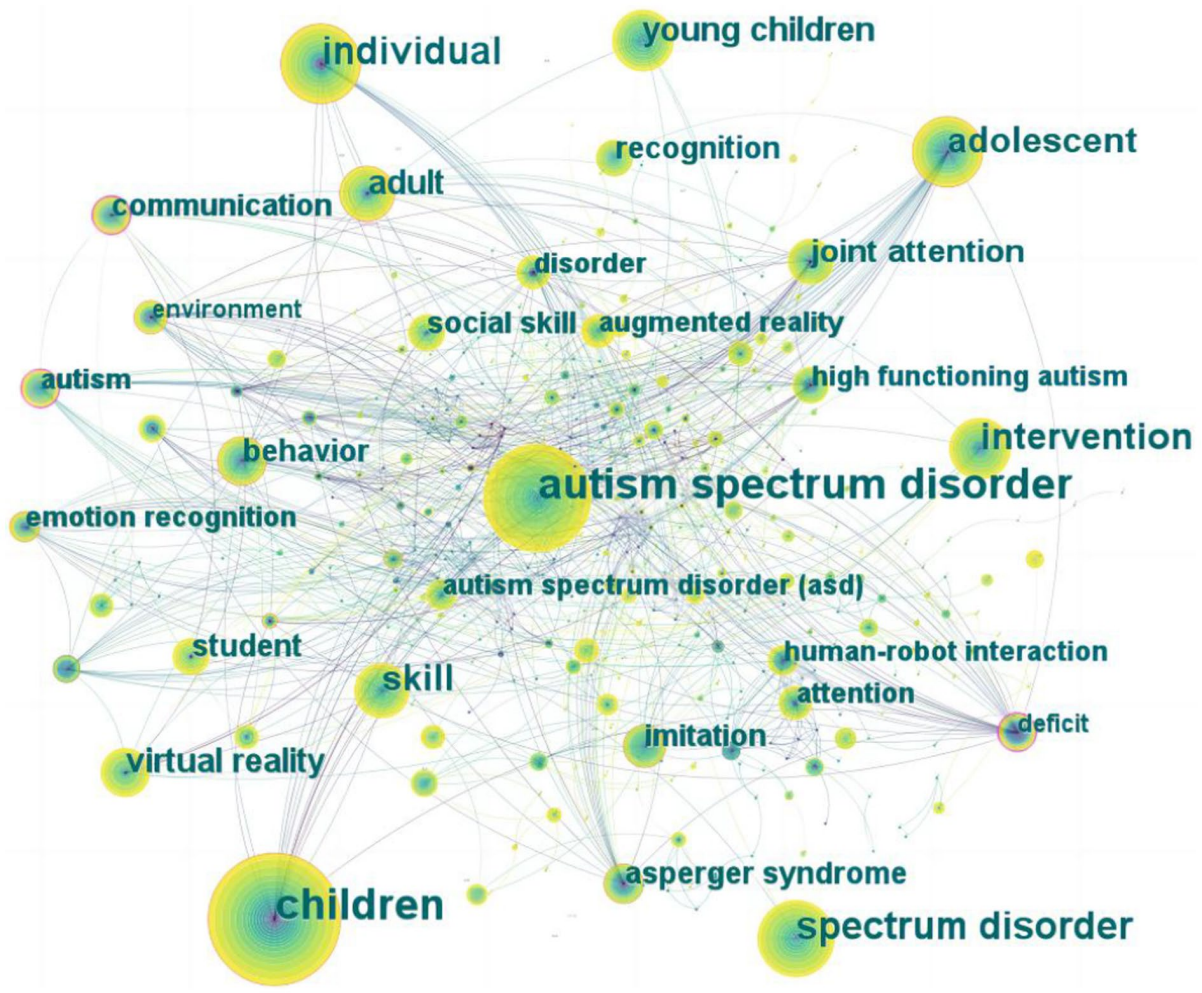
The co-occurrence network of keywords related to DTx on ASD.

A network map of keywords was generated, consisting of 425 nodes and 1704 links (Figure 7). A total of 425 research keywords were identified, revealing the most common topics. Based on frequency and centrality (Supplementary Table 4), the most popular keywords were “autism spectrum disorder,” “children,” “individual,” and “intervention.”

The top 10 keywords with the highest occurrence frequency and centrality were selected to help understand the research hotspots in the field from 2002 to 2022. “Autism spectrum disorder” was the most frequent keyword (174), followed by “children” (173) and “individual” (85). “Deficit” had the highest centrality (0.4), followed by “communication” (0.23) and “emotion recognition” (0.2; Figure 7; Supplementary Table 4).

**Figure 8:**
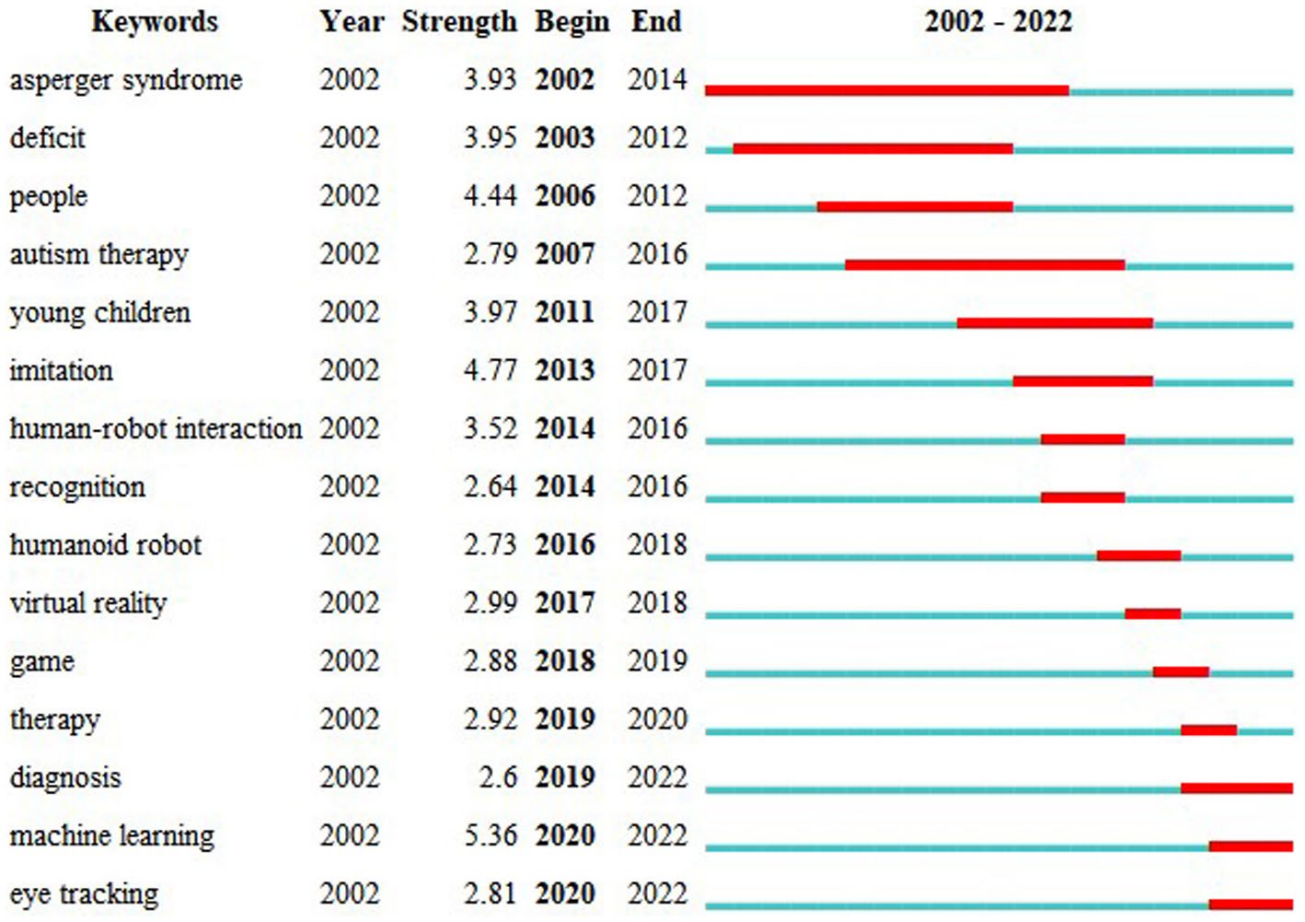
Top 15 keywords with the strongest citation bursts.

The top 15 most-cited keywords are shown in Figure 8. The keyword “machine learning,” which appeared starting in 2020, showed the strongest citation burst of 5.36.

**Figure 9:**
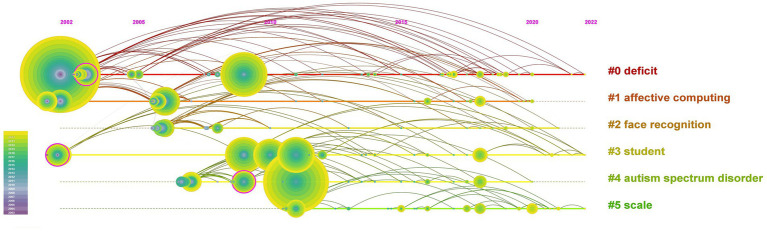
The timeline views of keywords.

To obtain a clearer image of the current research subjects involving DTx in the field of ASD, we clustered, analyzed, and summarized these terms. The grouping was reasonable and relevant with a Q value of 0.7378 and an S value of 0.8864 after clustering. A total of 18 clusters were generated to reflect topical trends, with the top six clusters of keywords being “deficit,” “affective computing,” “face recognition,” “student,” “autism spectrum disorder,” and “scale” (Supplementary Figure 7). The timeline view in Figure 9 shows the six clusters.

## 4. Discussion

The use of DTx in ASD is developing rapidly and gaining the attention of researchers worldwide, especially in the United States. Both Vanderbilt University and Yale University are significant institutions in the field for their long-term research, leading the direction of ASD research to some extent. The researcher from Vanderbilt University, Warren, Zachary, has published several academic publications in the field, drewing more attention from his peers. In addition, the application of new technologies such as VR, ML, and eye-tracking in this field has driven the development of DTx on ASD and is currently a popular research topic. This paper summarizes the current global research status of DTx in ASD, and explores future research trends.

As can be seen from the annual article publication volume, it can be roughly divided into three periods. During the first period (2002–2010), the number of publications was low. This rapidly increased from the second period (2010–2020), with a fluctuation in the publication numbers. This might be due to an increased interest in this field by scholars and the vigorous development of AI during this period (35). In addition, COVID-19 has led to an increased prevalence of public mental illness (37). The FDA has released a number of interim policies to support digital health innovations during the COVID-19 pandemic, such as expanding guidance documents on the use of DTx for the treatment of mental illness (38), thus stimulating the development of DTx. The third period was from 2020 to 2022, which showed a decreased overall growth rate. According to the macro trend analysis, research on DTx for ASD may have a slightly slower overall growth trend in the coming years. This is possibly due to some problems in the application and commercialization of DTx (39, 40). Therefore, although DTx has made great progress in its applications for ASD, it still needs time to be broadly implemented.

Most publications originated from the United States, followed by the United Kingdom. This can be explained by their economic and policy support. As a developed country with cutting-edge technology, the publications in the United States are far ahead of other countries. The growth of DTx in the field of ASD has been aided by the promotion of United States policies and increased attention to ASD in children. The FDA and the Radiation Health Center established “digital health plans” to advance public health and support digital health users. They also created and implemented regulatory strategies and policies for digital health technologies (41), which significantly sped up the approval and marketing of DTx technology. ASD research is heavily backed financially and socially in the United States. From 2007 to 2010, legislation requiring insurance companies to cover ASD treatment and interventions was passed in a number of American states (42). Furthermore, the Americans with Disabilities Act and other legislation passed by the United States include provisions pertaining to the rights and interests of people with ASD (43). Additionally, federal funding organizations and foundations together allot billions of dollars each year to support biomedical research in the United States (43). England has been active in the field of DTx for ASD. It has proposed the establishment of a database of children with ASD in 2009 (36) and introduced reimbursement plans in 2019 (44). Therefore, other countries can also provide more relevant policy and economic support to promote DTx research on ASD.

Most publications and cooperative network, respectively, originated from Vanderbilt University and Yale University. This finding can be explained by their historical deposition and scientific research emphasis in this field. The first center for ASD and innovation in the world, Vanderbilt University, aims to invent and commercialize new technologies to improve the quality of life of people with ASD. It also used VR systems to assess social communication and collaboration in ASD (34, 45). To avoid discriminating against physical health, Vanderbilt University used the National Institutes of Health RePORTER to systematically review funded studies on all physical health disparities in adults with ASD. The findings suggested that new policies are needed to support research on developmental or mental health disparities (46). Yale University is the only university with centrality, indicating that it is the main research institution in this field and the core of complex networks. The Yale Child Study Center, which serves as the Department of Child Psychiatry for the Yale School of Medicine, has been serving families through the integration of evidence-based clinical practice, training, and research for over 100 years. Yale University’s research focuses on the early social and emotional development of ASD and has also used VR behavioral interventions for ASD (47, 48).

Warren, Zachary is a scientific researcher at Vanderbilt University among the top five high-achieving authors, who is committed to the forefront of ASD research with a high *H*-index from 2017 to 2018. His research at this stage mainly focused on the use of VR to assess the functional performance of children with ASD and enhance social abilities (49, 50). Warren, Zachary et al. (51) also systematically evaluated a novel mobile-health screening tool for ASD in 2018, demonstrating the utility of this product. This also set the stage for the first FDA-certified ASD diagnostic product produced by Cognoa in 2021 (11).

Through the clustering of references, we found that articles related to VR were highly cited, showing that it has been a research hotspot in the field of DTx on ASD. The significant advantage of VR is that it can simulate various social situations (52) and has the characteristics of individuation (53), which is critical for some skills training on ASD. Manju et al. (54) proposed VR-based therapy to improve social ability, emotion, and attention in children with ASD, which actually explains the characteristics of individuation, immersion and multi-sensory experience, playing an important part in DTx. Therefore, DTx combined with VR can make up for the traditional method that some treatment schemes and scenarios cannot be replicated, providing a more realistic and diversified treatment environment for ASD.

The most common recent keywords were “machine learning” and “eye tracking,” which may represent future research trends and direction. As the most common word in citations, “machine learning” has great potential in enhancing diagnostic and intervention research in behavioral science and may be particularly useful in investigations involving ASD (55). In order to automatically predict ASD in young children, Kojovic et al. (56) employed 2D films based on ML, and their model had an 80.9% accuracy rate. Furthermore, Prefontaine et al. (57) compared 5 ML algorithms to detect ASD in children and discovered that each calculation was superior to random sampling, indicating that ML is also a promising method that can be used to estimate the prognosis of kids with ASD and help practitioners. There is still room for ML to grow. Eye-tracking is an important tool within the AI field. Eye-tracking technology is digital, and its numerous paradigms can read facial expression data for autistic children (58); DTx based on this is also a subject of study. The combination of eye-tracking, visualization, and ML offers significant potential for creating objective tools to help with ASD screening, according to a study on eye-tracking by Cilia et al. (59).

## 5. Limitations

For the purpose of conducting a comprehensive bibliometric analysis of literature relating to DTx in ASD, this study established a time frame from 2002 to 2022. However, there are certain limitations with this study that must be addressed. First, we only analyzed English articles in the WoS database, which may lead to language and publication bias. Second, although the inclusion conditions limited the number of years, the year of the cited articles is still comprehensive, and the data may be misleading.

## 6. Conclusion

This study revealed the current state of research on DTx for the diagnosis, management, and treatment of ASD as well as the popular topics and research frontiers. DTx are evolving rapidly. VR, ML, and eye-tracking have become popular technologies in this field. More cross-regional and cross-disciplinary collaborations are recommended to advance the development and availability of DTx.

## Data availability statement

The original contributions presented in the study are included in the article/Supplementary material, further inquiries can be directed to the corresponding author.

## Author contributions

XW and HD contributed to study conception, design, and manuscript revision. XW, SJ, HC, QL, and RG contributed to data collection, analysis, and manuscript writing. JW contributed to obtaining funding and manuscript revision. All authors contributed to the article and approved the submitted version.

## Funding

This study was supported by the Guiding Project of Fujian Provincial Department of Science and Technology, China (Grant no. 2022Y0038).

## Conflict of interest

The authors declare that the research was conducted in the absence of any commercial or financial relationships that could be construed as a potential conflict of interest.

## Publisher’s note

All claims expressed in this article are solely those of the authors and do not necessarily represent those of their affiliated organizations, or those of the publisher, the editors and the reviewers. Any product that may be evaluated in this article, or claim that may be made by its manufacturer, is not guaranteed or endorsed by the publisher.
