# Supplementary material for: Global trends and hotspots in the digital therapeutics of autism spectrum disorders: a bibliometric analysis from 2002 to 2022
Source: Front Psychiatry. 2023 May 15;14:1126404. doi: 10.3389/fpsyt.2023.1126404 (PMC10225518; doi:10.3389/fpsyt.2023.1126404)
Supplement: Supplementary file 1 [file Data_Sheet_1.docx]

Supplementary Material

**Global Trends and Hotspots in the Digital Therapy of Autism Spectrum Disorders: a bibliometric analysis from 2002 to 2022**

**Xuesen Wu^1†^, Haiyin Deng^1†^, Shiyun Jian^1^, Huian Chen^1^, Qing Li^1^, Ruiyu Gong^1^, Jingsong Wu^1,2*^**

^1^ College of Rehabilitation Medicine, Fujian University of Traditional Chinese Medicine, Fuzhou, Fujian, China.

^2^ Innovation and Transformation Center, Fujian University of Traditional Chinese Medicine, Fuzhou, Fujian, China.

^†^These authors contributed equally to this work and share first authorship.

*** Correspondence:**

Jingsong Wu

jingsongwu01@163.com

# Supplementary Figures

## Flow chart of bibliometrics analysis.


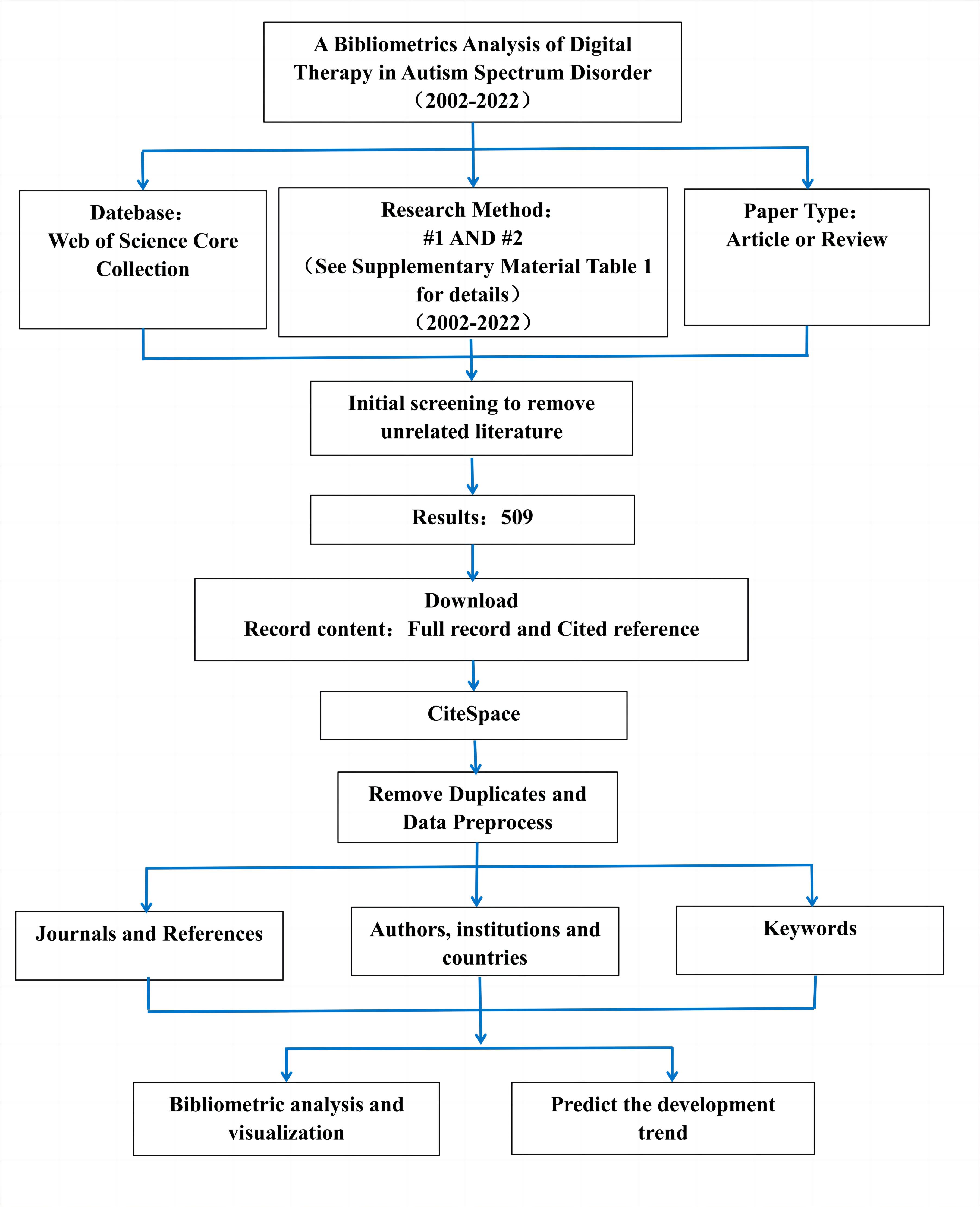


**Figure 1** A frame flow diagram showing the detailed selection criteria and bibliometric analysis steps of DTx on ASD. SCI-E, Science citation index expand.

- 1. **Pie chart of document type.**

For the document types in the articles, there are 451 articles (89%), and 58 review articles (11%).


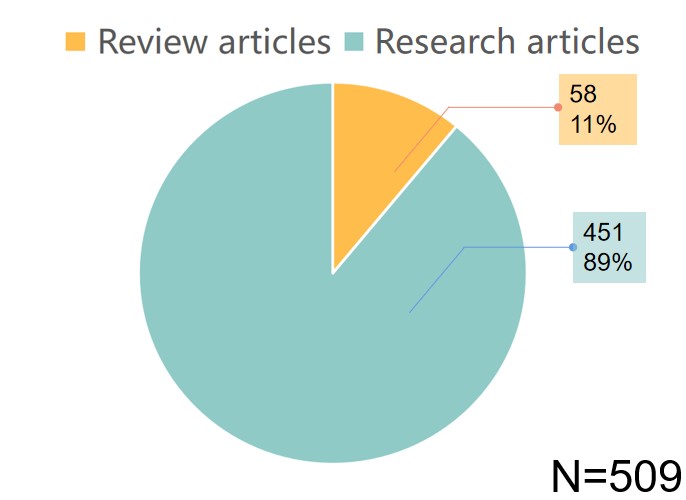


**Figure 2.** The number and percentage of each kind of document types related to DTx on ASD.

## The co-occurrence map of cited journals.

In order to visually analyze the relevant data of cited journals and explore the possible potential correlation, CiteSpace software was used to draw the co-occurrence map of cited journals with centralized representation. The result shows that the top five journals with centrality were *Child Development* (0.39), *Archives of General Psychiatry* (0.22), *Acta psychiatrica Scandinavica* (0.22), *BRAIN* (0.2), *British Journal of Psychiatry* (0.15).


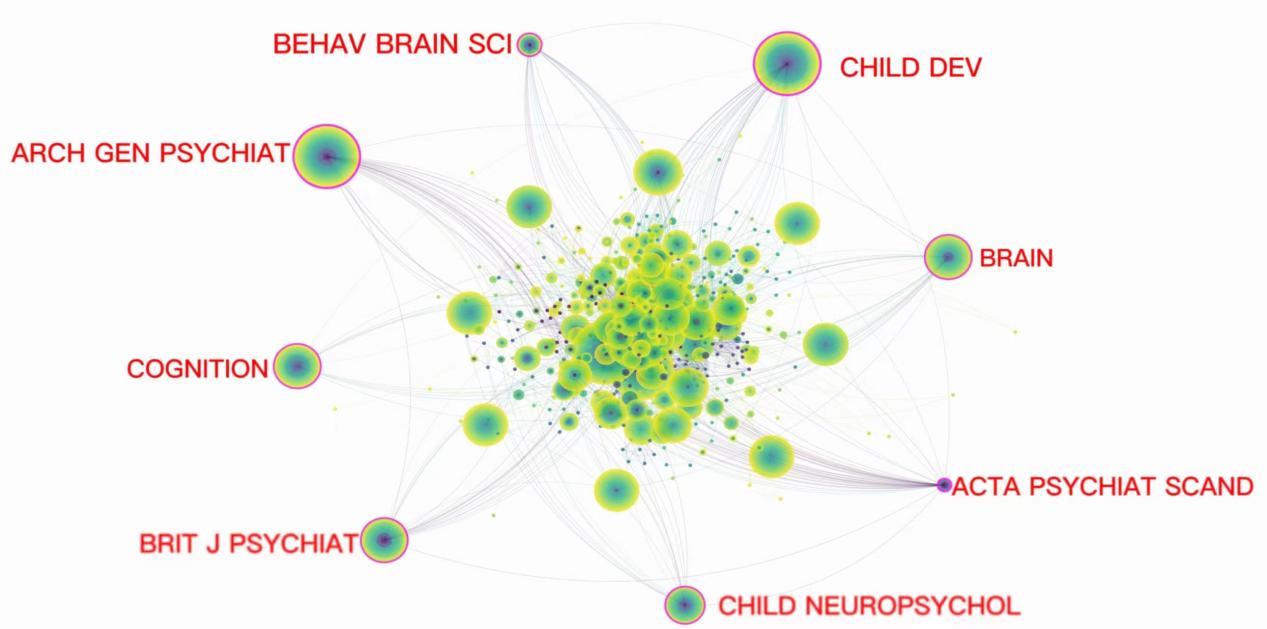


**Figure 3.** The citation network of journals related to DTx on ASD.

## The histogram of total citations by top five authors.

To visually compare the citations of the top five authors with most publications, a histogram was plotted as follows. The results show that the articles from Sarkar, Nilanjan and Warren, Zachary are cited by more people. The total citation of articles they published is 511 and 456, respectively.


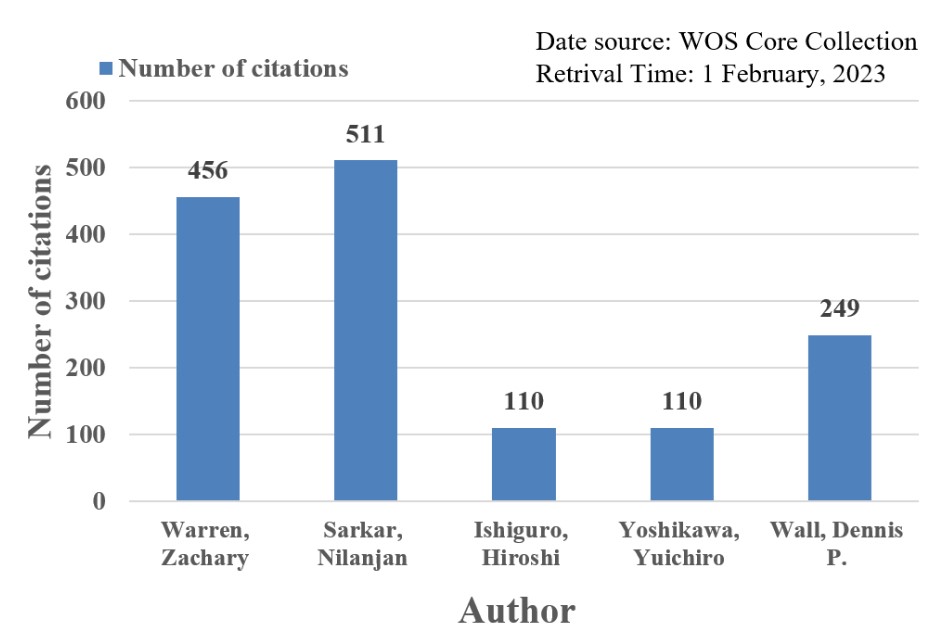


**Figure 4.** The number of citations of top five authors.

## The histogram of H-index per 2-year by top five authors.

In order to visually compare the academic influence of the top five authors with most publications, a histogram was plotted as follows. Among them, Warren, Zachary had the highest H-index in 2017-2018, at 7. It shows that Warren, Zachary's academic achievements in these two years were more outstanding and had more reference value.


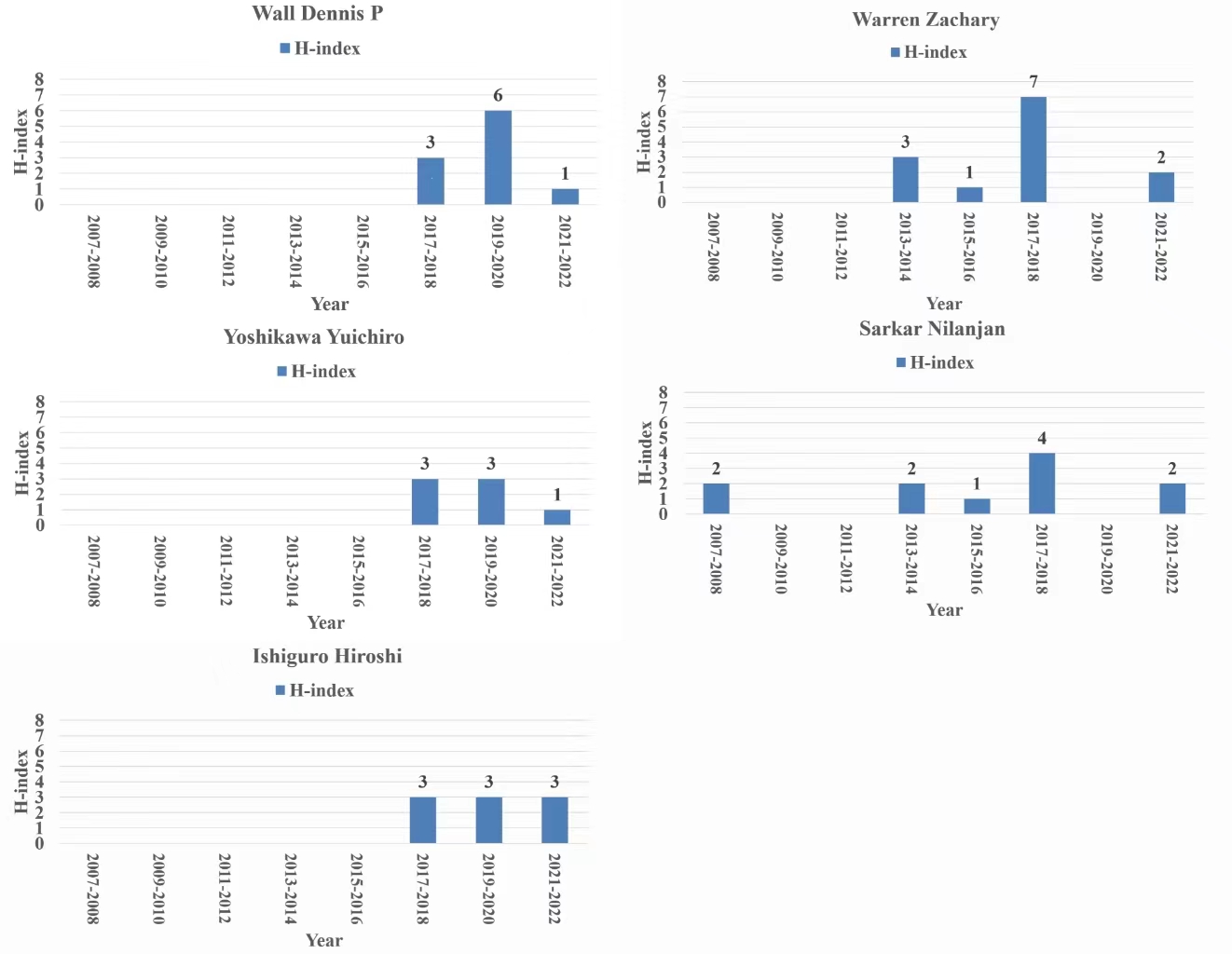


**Figure 5.**  The H-index per 2-year of the top five authors from 2002-2022.

## The co-occurrence map of the references.

To visually analyze the relevant data of the references and explore the possible potential correlation, CiteSpace software was used to draw the co-occurrence map of the references with centralized representation. The result shows that the top five articles with frequency were published by American Psychiatric Association in 2013 (86), Scassellati, Brian in 2012 (73), Diehl, Joshua J in 2012 (67), Catherine Lord in 2000 (42), and John - John Cabibihan in 2013 (42). In these several with the highest cited references, there is no purple ring, indicating that their centrality is insufficient and the connection is not close enough.


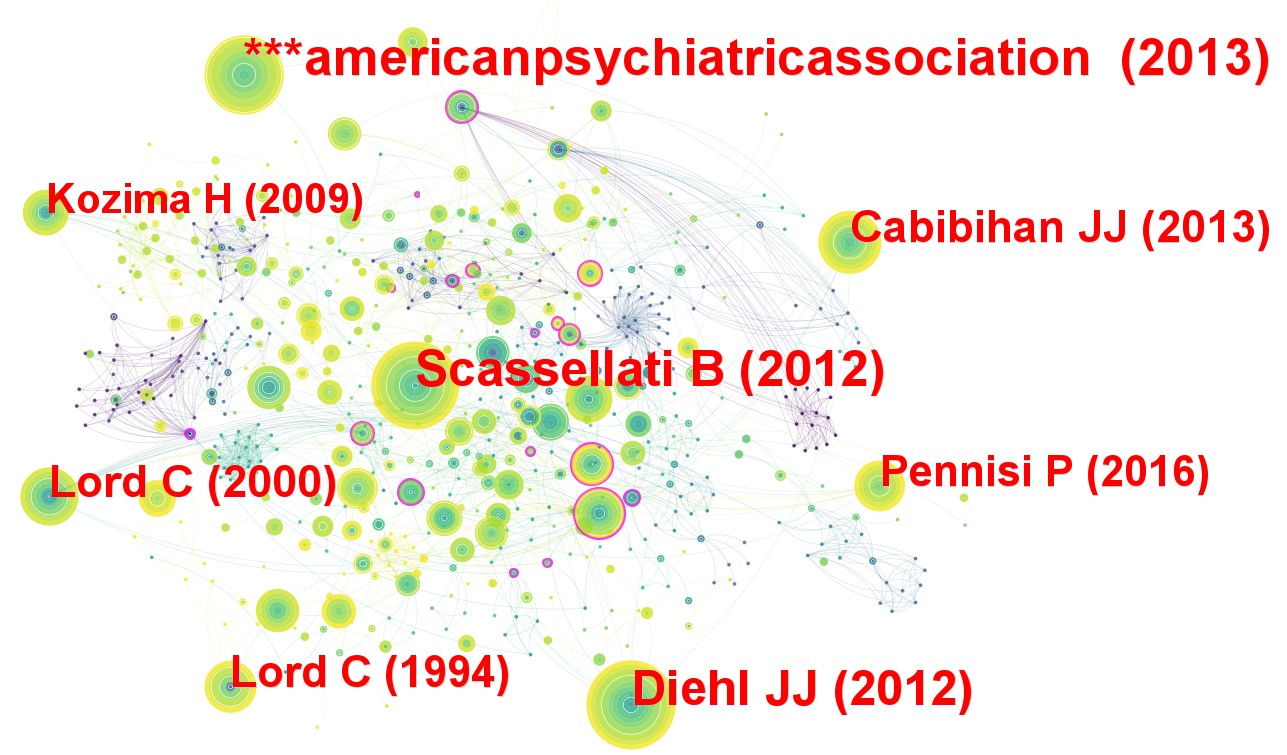


**Figure 6** The citation network of reference related to DTx on ASD.

## Flow chart of bibliometrics analysis.

In order to get a clearer knowledge of the current research subjects linked to DTx on ASD, CiteSpace software was used to draw the cluster map of keywords. The top six clusters of keywords are "deficit", "affective computing", "face recognition", "student", "autism spectrum disorder" and "scale".


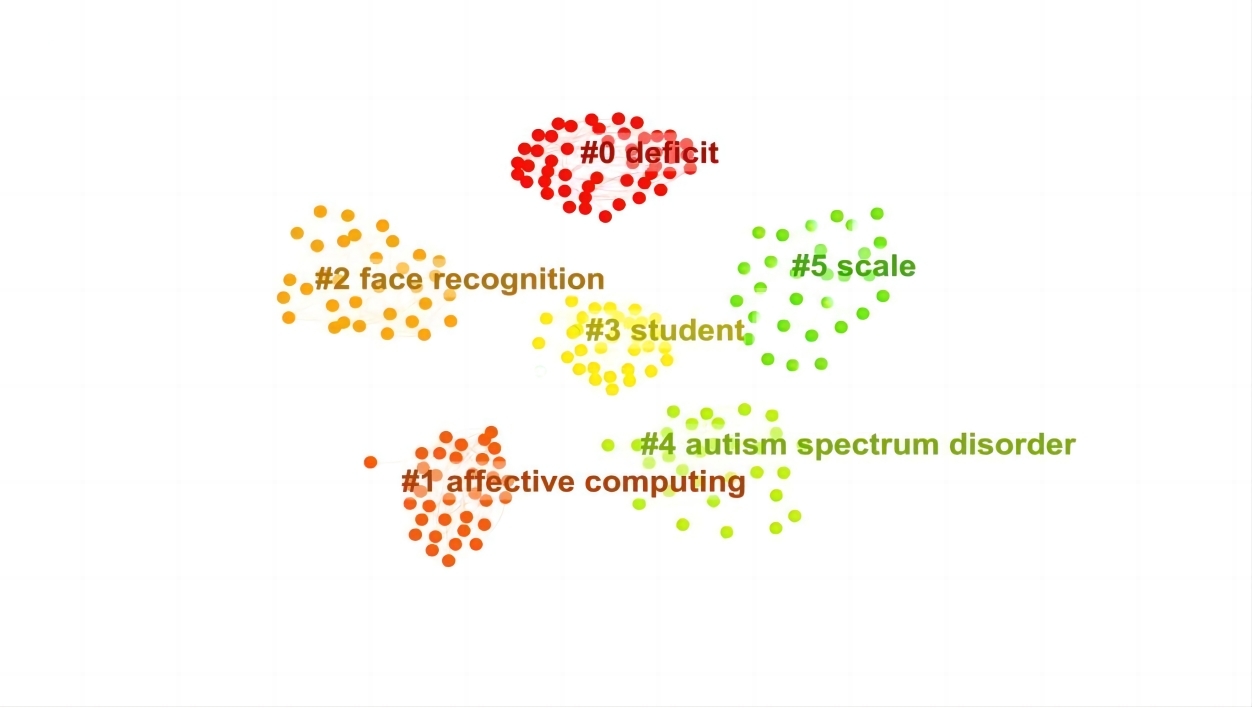


**Figure 7** The cluster map of keywords related to DTx on ASD.

1. **Supplementary Tables**
   1. **Search queries.**

**Table 1** Search queries.

| **Set** | **Result** | **Search Query** |
| --- | --- | --- |
| #1 | 71625 | ( TS=（ autism spectrum disorder） OR TS=（asperger syndrome） OR TS=（autistic disorder） OR TS=（child development disorders） OR TS=（ autis*） OR TS=（ pervasive developmental disorder） OR TS=(asperger*) OR TS=(rett syndrome) OR TS=(childhood disintegrative disorder) OR TS=(disintegrative psychosis) OR TS=(heller syndrome) OR TS=（ASD） OR TS=(PDD) OR TS=( PDD-NOS) )  Indexes=Web of Science Timespan=2002-2022 |
| #2 | 10886111 | （TS=(digital*) OR TS=(software) OR TS=（software*） OR TS=（video game） OR TS=( computer*) OR TS=（virtual reality） OR TS=（VR） OR TS=（robot*） OR TS=（augmented reality） OR TS=（Computer Assisted） OR TS=（man-machine systems） OR TS=（telemedicine） OR TS=（tele rehabilitation） OR TS=(computer simulation) OR TS=(user-computer interface) OR TS=(video games) OR TS=( microcomputers) OR TS=(electronics) OR TS=( robotics) OR TS=(smartphone) OR TS=（smartphone*） OR TS=（ipad） OR TS=（laptop） OR TS=(mobile applications) )  Indexes=Web of Science Timespan=2002-2022 |
| #3 | 3195 | #1 AND #2 |

- 1. **Descriptive statistics of the bibliometrics from the top-10 cited journals.**

**Table 2** Descriptive statistics of the bibliometrics from the top-10 cited journals.

| **Bibliometrics** | **Median** | **25** | **75** | **IQR** |
| --- | --- | --- | --- | --- |
| IF | 4.489 | 3.765 | 7.870 | 4.105 |
| Eigenfactor | 0.0143 | 0.0082 | 0.0221 | 0.0138 |
| CiteScore | 6.750 | 5.850 | 9.825 | 3.975 |
| SNIP | 2.006 | 1.560 | 2.944 | 1.384 |
| SJR | 1.292 | 0.949 | 2.310 | 1.361 |

- 1. **Top 10 frequency and centrality of cited authors.**

To show the academic level of the cited authors more intuitively, we made the following table. The results show that Baron-cohen S has the highest centrality of the cited authors at 0.62（0.33＋0.29）, and Lord Catherine has the highest frequency at 152.

**Table 3** Top 10 frequency and centrality of cited authors related to DTx on ASD.

| **Rank** | **Frequency** | **Author** | **Year** | **Centrality** | **Author** | **Year** |
| --- | --- | --- | --- | --- | --- | --- |
| 1 | 152 | Lord C | 2002 | 0.35 | Billard A | 2005 |
| 2 | 97 | Robins B | 2008 | 0.33 | Baron-cohen S | 2002 |
| 3 | 93 | Baron-cohen S | 2002 | 0.29 | Baroncohen S | 2002 |
| 4 | 91 | Scassellati B | 2008 | 0.24 | Dawson G | 2002 |
| 5 | 82 | Dawson G | 2002 | 0.18 | Klin A | 2002 |
| 6 | 68 | Kozima H | 2008 | 0.16 | Baron-cohen SIMON | 2006 |
| 7 | 68 | Diehl JJ | 2013 | 0.14 | Anderson CJ | 2011 |
| 8 | 59 | Dautenhahn K | 2007 | 0.13 | Howlin P | 2012 |
| 9 | 50 | Parsons S | 2005 | 0.12 | Parsons S | 2005 |
| 10 | 50 | Rutter M | 2008 | 0.12 | Ruble LA | 2008 |

- 1. **Top 10 frequency and centrality of keywords.**

To better evaluate the frontier themes or emerging trends in the ASD field, a table was made as follows. The results show that "autism spectrum disorder" is the highest-frequency keyword with a frequency of 174. And "defect" is the highest-centrality word at 0.4, the second is "communication" at 0.23, and the third is "emotion recognition" at 0.2, indicating that these are also the important research contents of DTx in the field of ASD.

**Table 4** Top 10 frequency and centrality of keywords related to DTx on ASD.

| **Rank** | **Keyword** | **Frequency** | **Rank** | **Keyword** | **Centrality** |
| --- | --- | --- | --- | --- | --- |
| 1 | autism spectrum disorder | 174 | 1 | deficit | 0.4 |
| 2 | children | 173 | 2 | communication | 0.23 |
| 3 | individual | 85 | 3 | emotion recognition | 0.2 |
| 4 | intervention | 68 | 4 | adolescent | 0.2 |
| 5 | adolescent | 57 | 5 | individual | 0.17 |
| 6 | skill | 56 | 6 | diagnostic interview | 0.17 |
| 7 | young children | 54 | 7 | imitation | 0.16 |
| 8 | virtual reality | 51 | 8 | asperger syndrome | 0.15 |
| 9 | adult | 47 | 9 | people | 0.15 |
| 10 | joint attention | 40 | 10 | autism therapy | 0.13 |
